# The nutritional phenome of EMT-induced cancer stem-like cells

**DOI:** 10.18632/oncotarget.2147

**Published:** 2014-06-30

**Authors:** Elisabet Cuyàs, Bruna Corominas-Faja, Javier A. Menendez

**Affiliations:** ^1^ Metabolism & Cancer Group, Translational Research Laboratory, Catalan Institute of Oncology, Girona, Catalonia, SPAIN; ^2^ Girona Biomedical Research Institute (IDIBGI), Girona, Catalonia, SPAIN

**Keywords:** Phenome, nutrients, metabolism, cancer, cancer stem cells, EMT

## Abstract

The metabolic features of cancer stem (CS) cells and the effects of specific nutrients or metabolites on CS cells remain mostly unexplored. A preliminary study to delineate the nutritional phenome of CS cells exploited the landmark observation that upon experimental induction into an epithelial-to-mesenchymal (EMT) transition, the proportion of CS-like cells drastically increases within a breast cancer cell population. EMT-induced CS-like cells (HMLER^shEcad^) and isogenic parental cells (HMLER^shCntrol^) were simultaneously screened for their ability to generate energy-rich NADH when cultured in a standardized high-throughput metabolic phenotyping platform comprising >350 wells that were pre-loaded with different carbohydrates/starches, alcohols, fatty acids, ketones, carboxylic acids, amino acids, and bi-amino acids. The generation of “phenetic maps” of the carbon and nitrogen utilization patterns revealed that the acquisition of a CS-like cellular state provided an enhanced ability to utilize additional catabolic fuels, especially under starvation conditions. Crucially, the acquisition of cancer stemness activated a metabolic infrastructure that enabled the vectorial transfer of high-energy nutrients such as glycolysis end products (pyruvate, lactate) and bona fide ketone bodies (β-hydroxybutyrate) from the extracellular microenvironment to support mitochondrial energy production in CS-like cells. Metabolic reprogramming may thus constitute an efficient adaptive strategy through which CS-like cells would rapidly obtain an advantage in hostile conditions such as nutrient starvation following the inhibition of tumor angiogenesis. By understanding how specific nutrients could bioenergetically boost EMT-CS-like phenotypes, “smart foods” or systemic “metabolic nichotherapies” may be tailored to specific nutritional CSC phenomes, whereas high-resolution heavy isotope-labeled nutrient tracking may be developed to monitor the spatiotemporal distribution and functionality of CS-like cells in real time.

During the past 30 years, cancer research has been dominated by molecular biology, which has drastically overshadowed other credible approaches in the field. Metabolism appears to be the most etiolated field under the large umbrella of gene-centered cancer research. Unsurprisingly, the unique metabolic signatures of cancer cells, which were recognized nearly a century ago by Otto Warburg [1-3], have been frequently perceived as evolutionary conserved targets that are programmed by oncogenic gain-of-function events and the loss of tumor suppressors [4-6]. In the last few years, however, provocative evidence has accumulated to suggest that metabolism is not simply a consequence; rather, it might play a pivotal role in dictating the different phenotypic states exhibited by heterogeneous cancer cell populations, including those referred to as cancer stem (CS) cells [7-13]. CS cells have been causally implicated as drivers of primary tumor growth and are involved in seeding and establishing metastasis from most human epithelial carcinomas. On the one hand, cancer is now understood to be a disease of cellular reprogramming that involves progressive resetting of the metabolic infrastructure and metabolite levels concomitantly with changes in cellular differentiation [7, 14]. On the other hand, the modulation of metabolism and the associated signaling pathways has been increasingly implicated in cell identity determination during cellular reprogramming and oncogenesis [15-20]. Crucially, a transformation in cellular metabolism appears to precede changes in stemness; therefore, metabolic reprogramming may reflect the molecular dynamics fundamental to cell fate rearrangement and redirection.

Recent studies have revealed that a metabolic switch to glucose metabolism is a critical promotional event in the epithelial-to-mesenchymal (EMT)-driven CS-like phenotype. Epigenetic silencing of the gluconeogenic enzyme fructose-1,6-biphosphatase, which catalyzes the energy-consuming conversion of fructose 1,6-biphosphate to fructose-6-phosphate, is employed by CS cells as a mechanism of glucose flux maintenance via glycolysis and other associated biosynthetic pathways [21]. An increased reliance on glucose metabolism, in turn, reduces the level of reactive oxygen species (ROS) to promote EMT and the CS-like phenotype. Glucose also appears to act as an essential nutrient for CS cells, as its presence in the culture environment significantly increases the percentage of CS-like cells in the overall cancer cell population [22]; in contrast, glucose starvation is sufficient to cause a rapid depletion of the CS-like subpopulation *in vitro*. However, the ultimate metabolic features of CS cells and the effects of specific nutrients on CS cells remain largely unexplored. Moreover, although the important effects of the tumor tissue niche on CS cells have been recognized in recent years, the impacts of key tumor microenvironmental nutrients on CS cells have not yet been determined.

We recently suggested that CS cells might possess specific metabolic properties that distinguish them from the majority of tumor cells and that such metabotypes may constitute a basis for new therapeutic strategies that metabolically eliminate CS cells [7, 23-25]. Herein, we hypothesized that a systematic evaluation of the phenotypic variations of non-CS cells and CS isogenic cell types in response to multiple carbon and nitrogen substrates may unambiguously define a preliminary nutritional phenome of CS cells in terms of their energy-producing metabolic pathways. Stable sibling cell lines in which an EMT had been induced to stably propagate CS-like-enriched populations [26, 27] were subjected to Phenotype MicroArrays for Mammalian Cells (PMM) (Biolog, Hayward CA, USA), a standardized platform for high-throughput metabolic phenotyping [28-32]. EMT-induced CS-like cells and isogenic control cells were simultaneously screened for the ability to generate energy-rich NADH when cultured in wells that had been pre-loaded with >350 different carbon-energy and nitrogen-based substrates, including carbohydrates/starches, alcohols, fatty acids, ketones, carboxylic acids, amino acids, and bi-amino acids. We present herein the first-in-class nutritional-phenomic map confirming that the enhancement of certain catabolic energy-producing pathways in CS-like cells should be considered a specific nutritional property that phenotypically distinguishes CS cells from non-CS cells. Crucially, we present the first evidence of the existence of a cell-autonomous “reverse Warburg effect” in EMT-driven CS-like cells, where reprogramming to a CS-like cellular state appears sufficient to allow a cell-autonomous, vectorial transfer of energy-rich nutrients from the extracellular microenvironment to the CS-like cells' energy-producing catabolic pathways.

## Nutritional phenome of non-starved, EMT-induced CS-like cellular states

We used experimentally transformed HMLER breast cancer cells (human mammary epithelial cells [HMECs] overexpressing hTERT, SV40 T/t and H-RasV12) that had been modified to inhibit expression of the human CDH1 (E-cadherin) gene *via* short hairpin RNA (shRNA; HMLER^shECad^ cells), which constitutes a valuable method for drastically enriching cells with CS-like properties [26, 27]. We simultaneously profiled these cells and the stable isogenic line HMLER^shCntrol^ in four microplates (termed PM-Ms) in which the bottoms of the wells had been coated with substrate nutrients to create 367 unique culture conditions. PM-M1 contained primarily carbohydrate and carboxylate substrates, whereas PM-M2, M3, and M4 contained individual L-amino acids and most dipeptide combinations. The PM assay was conducted during a 2-day incubation period, and the HMLER^shCntrol^ and HMLER^shECad^ cells were incubated in Biolog IF-M1 medium (RPMI 1640 without glucose/glutamine; this medium provided all nutritional ingredients at sufficient levels other than major C- and N-sources, which were omitted) containing 5% serum.

**Figure 1:**
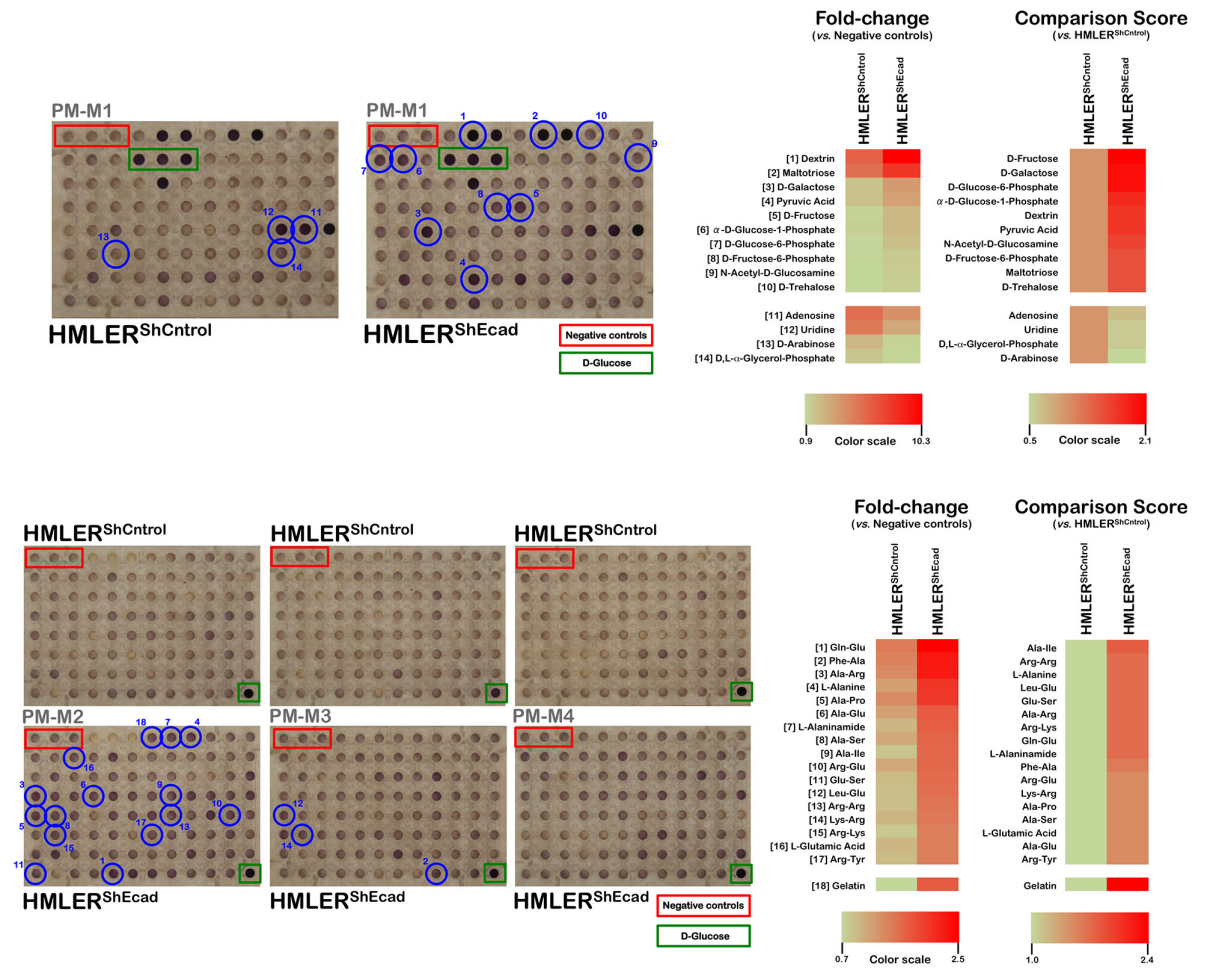
Metabolic fingerprint of non-starved, EMT-induced CS-like cellular states *Right panels.* 50 μL per well of 400,000 cells/mL suspensions of non-CS HMLER^shCntrol^ and CS-like HMLER^shECad^ cells (20,000 cells per well) in Biolog IF-M1 medium, *i.e.,* RPMI-1640 medium that lacked phenol red and depleted of carbon-energy sources (no glucose, low glutamine [0.3 mmol/L] and low FBS [5%]), were inoculated into Phenotype MicroArrays PM-M1 through PM-M4 (Biolog, Hayward, CA) which contained 367 biochemical substrates that could potentially be metabolized and provide energy for cells. After 48 h incubation in RPMI-1640 and glucose and was supplemented with penicillin/streptomycin and reduced levels of glutamine [0.3 mmol/L] and FBS, plates were incubated at 37 °C under air to assess dye reduction 6 h (Redox Dye Mix MA) and then photographed. This 2-days incubation should allow cells to use up residual carbon-energy sources in the 5% serum (*e.g.,* 5% serum would contribute about 0.35 mmol/L glucose, plus lipids, and amino acids) and minimizes the background color in the negative control wells, which have no added biochemical substrate [30]. Furthermore, the 2-days incubation should allow cells to transition their metabolism to use the various substrates provided in the wells. The respective utilization of substrates to generate energy-rich NADH was measured as ODs at 590 nm. Negative controls (red boxes) have no substrate in the well. Wells containing D-glucose (green boxes) serve as positive controls. Thresholds were set to disregard small and insignificant changes, and all of the wells that exceed this threshold are marked with blue circles to denote differentially metabolized substrates for each cell line, which are described in the text. Parallel experimentation using conventional 96-well plates was performed using the MTT redox dye to ensure that the 2-days incubation with Biolog IF-M1 medium failed to significantly alter the baseline cell growth of non-CS HMLER^shCntrol^ and CS-like HMLER^shECad^ cells (data not shown). Importantly, whereas widely employed redox dyes such as MTT, MTS or XTT measure nonspecific cellular reductase activities, the redox chemistry employed in the PMM technology give very little non-specific dye reduction and are reduced in a manner that is strictly dependent on the presence of usable carbon-energy sources in the medium. Therefore, this redox dye approach accurately measures reductase activity due to energy- (*i.e.,* NADH) producing catabolic pathways that use diverse biochemical substrates. *Left panels.* Phenetic maps of carbon and nitrogen utilization patterns of non-CS HMLER^shCntrol^ and CS-like HMLER^shECad^ cells under non-starved conditions. Phenotypes that are lost are colored green and phenotypes that are gained are colored red; the exact relative values are given by a corresponding color as indicated at the color scales.

**Figure 2:**
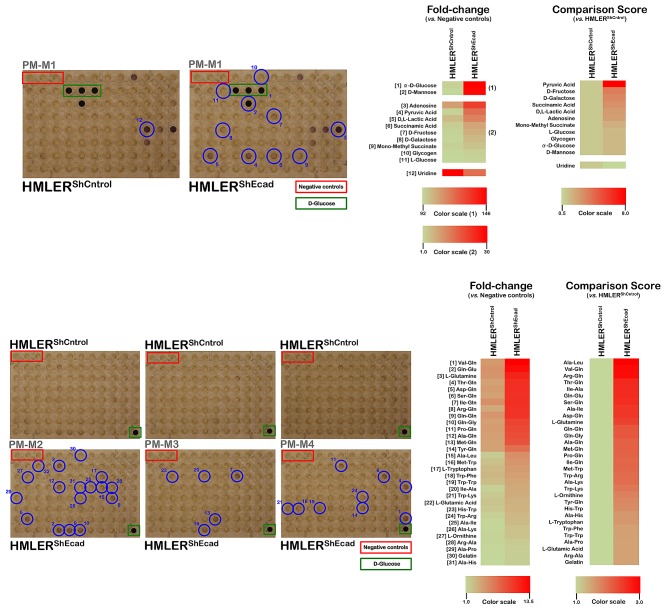
Metabolic fingerprint of the starved EMT-induced CS-like cellular state *Right panels.* Experiments were conducted as in Figure 1 with the exception of the culture medium employed, which lacked serum, glucose, and amino acids. *Left panels.* Phenetic maps of carbon and nitrogen utilization patterns of non-CS HMLER^shCntrol^ and CS-like HMLER^shECad^ cells under starvation conditions. Phenotypes that are lost are colored green and phenotypes that are gained are colored red; the exact relative values are given by a corresponding color as indicated at the color scales.

Because the color formed from each substrate reflected the energy-producing activity of the associated catabolic pathway, it was clear that non-CS HMLER^shCntrol^ and CS-like HMLER^shEcad^ cells both exhibited strong reductive responses in wells containing D-glucose (Fig. 1 and Fig. 2; green boxes [positive controls], all panels) and little or no response in wells lacking any carbon source (Fig. 1 and Fig. 2; red boxes [negative controls], all panels). To quantitatively compare each state rapidly and systematically, we developed a scoring system based on the fold change in the optical density of each substrate at 590 nm (purple color) resulting from the accumulation of reduced dye over a 6-hour period after normalization of the values to those of the negative-control wells included in each of the PM-M plates. To quantify these comparisons, we also calculated a comparison score from the absolute ratio between the metabolic flows of the non-CS and CS-like cells upon comparison at the same time point (6 h).

Most changes that occurred following the acquisition of a CS-like cellular state (28 out of 31, 90%) were increases in the ability to generate energy from a higher extracellular nutrient level. Quantitatively, the greatest difference (2.1-fold) between the non-CS HMLER^shCntrol^ and CS-like HMLER^shEcad^ cells was observed for the ability of CS-like HMLER^shEcad^ cell populations to metabolize monosaccharides such as D-fructose and D-galactose (Fig. 1, *top panels*). We observed higher sugar phosphate metabolism (D-glucose-6-phosphate, α-D-glucose 1-phosphate, and D-fructose-6-phosphate) by CS-like HMLER^shEcad^ cells. CS-like HMLER^shEcad^ cells also exhibited increased metabolism of the amide between glucosamine and acetic acid (N-acetyl-D-glucosamine), a monosaccharide glucose derivative. The metabolism of disaccharides (D-trehalose), trisaccharides (maltotriose), and mixtures of D-glucose (dextrin) polymers also increased in CS-like HMLER^shEcad^ cells. Interestingly, the ketone pyruvic acid, the end product of glycolysis, was a substrate for which dye reduction significantly increased in CS-like HMLER^shEcad^ cells relative to the basal metabolic flows observed in HMLER^shCntrl^ cells in all cases.

There were only three carbon sources to which CS-like HMLER^shEcad^ cells exhibited a decreased catabolic response relative to the HMLER^shCntrl^ cells. CS-like HMLER^shEcad^ cells were slightly less efficient than HMLER^shCntrl^ cells in the catabolism of D, L-α-glycerol-3-phosphate, an intermediate common to both the lipid and carbohydrate metabolic pathways. CS-like HMLER^shEcad^ cells also exhibited reduced uridine metabolism and notably reduced by half the ability to catabolize the monosaccharide D-arabinose relative to HMLER^shCntrl^ cells (Fig. 1, *top panels*).

Despite a low absolute rate of dye reduction, CS-like HMLER^shEcad^ cells demonstrated enhanced catabolic responses to the amino acids alanine, glutamic acid, and arginine in addition to the dipeptides containing these amino acids (Fig. 1, *bottom panels*). Notably, whereas HMLER^shCntrl^ cells completely failed to produce energy from gelatin, a mixture of peptides and proteins produced by partial collagen hydrolysis, CS-like HMLER^shEcad^ cells efficiently used gelatin as a catabolic substrate to generate energy-rich NADH.

## Nutritional phenome of the starved EMT-induced CS-like cellular state

Cell metabolism under standard culture conditions may differ significantly from *in vivo* conditions because the cancer cell microenvironment is generally characterized by poor blood perfusion, hypoxia, and nutrient limitations. To evaluate how the cell-autonomous changes in nutrient utilization observed in EMT-induced CS-like cells would be affected by the stressful conditions of the tumor microenvironment, we partially recapitulated this specific metabolic milieu by re-testing the abilities of CS-like HMLER^shEcad^ cells and non-CS HMLER^shCntrol^ parental cells to produce energy-rich NADH from the same panel of 367 substrate nutrients in serum-, glucose-, and amino acid-free Biolog IF-M2 medium (RPMI 1640 without glucose/glutamine/amino acids) (Fig. 2). As the cell response to the nutrients over a 2-day incubation period mostly comprised rapid cell death, similar to the responses observed in wells with no substrates, this assay format can be considered to evaluate cell survival responses under different nutrient supply conditions.

Similarly to non-starved conditions, most of the changes that occurred following the acquisition of a CS-like cellular state upon starvation comprised increases in the ability to generate energy from a broader range of extracellular nutrients (42 out of 43, 98%). Remarkably, the greatest quantitative difference was in pyruvic acid metabolism, wherein starved CS-like HMLER^shEcad^ cells exhibited a metabolic level 8.0-fold higher than that observed in starved non-CS HMLER^shCntrol^ cells (Fig. 2, *top panels*). Moreover, pyruvic acid was not the sole glycolysis end product efficiently metabolized by the starved CS-like HMLER^shEcad^ cells; D, L-lactic acid, the most common accumulated acidic waste in the extracellular tumor tissue microenvironment, was efficiently utilized as an energetic fuel 2.6-fold faster in starved CS-like HMLER^shEcad^ cells than in starved non-CS HMLER^shCntrol^ cells. In response to starvation, the CS-like HMLER^shEcad^ cell populations exhibited a further increase in the ability to metabolize the monosaccharides D-fructose and D-galactose in addition to D-glucose. The metabolism of succinamic acid and its monomethyl ester was notably increased in starved CS-like HMLER^shEcad^ cells relative to starved HMLER^shCntrl^ cells. Intriguingly, the energy-generating capabilities of starved CS-like HMLER^shEcad^ cells appeared to not be restricted to D-sugars, as these cells were able to generate energy-rich NADH in a minimal medium that contained L-glucose as the sole carbon source. Starved CS-like HMLER^shEcad^ cells also significantly gained the ability to use the purine nucleoside adenosine as an energy-generating metabolic substrate (2.5-fold increase relative to starved HMLER^shCntrl^ cells).

Interestingly, as observed in the non-starved CS-like HMLER^shEcad^ cells, starved CS-like HMLER^shEcad^ cells reduced by half their ability to catabolize the nucleoside base uridine relative to starved HMLER^shCntrl^ cells (Fig. 2, *top panels*).

Starved CS-like HMLER^shEcad^ cells notably acquired the ability to catalyze glutamine and multiple glutamine-containing dipeptides (*e.g.*, 13) to generate energy-rich NADH (Fig. 2, *bottom panels*). Starved CS-like HMLER^shEcad^ cells additionally gained the ability to catabolize the amino acid tryptophan and tryptophan-containing dipeptides.

## EMT-induced CS-like cells strongly catabolize exogenous mitochondrial fuel

**Figure 3:**
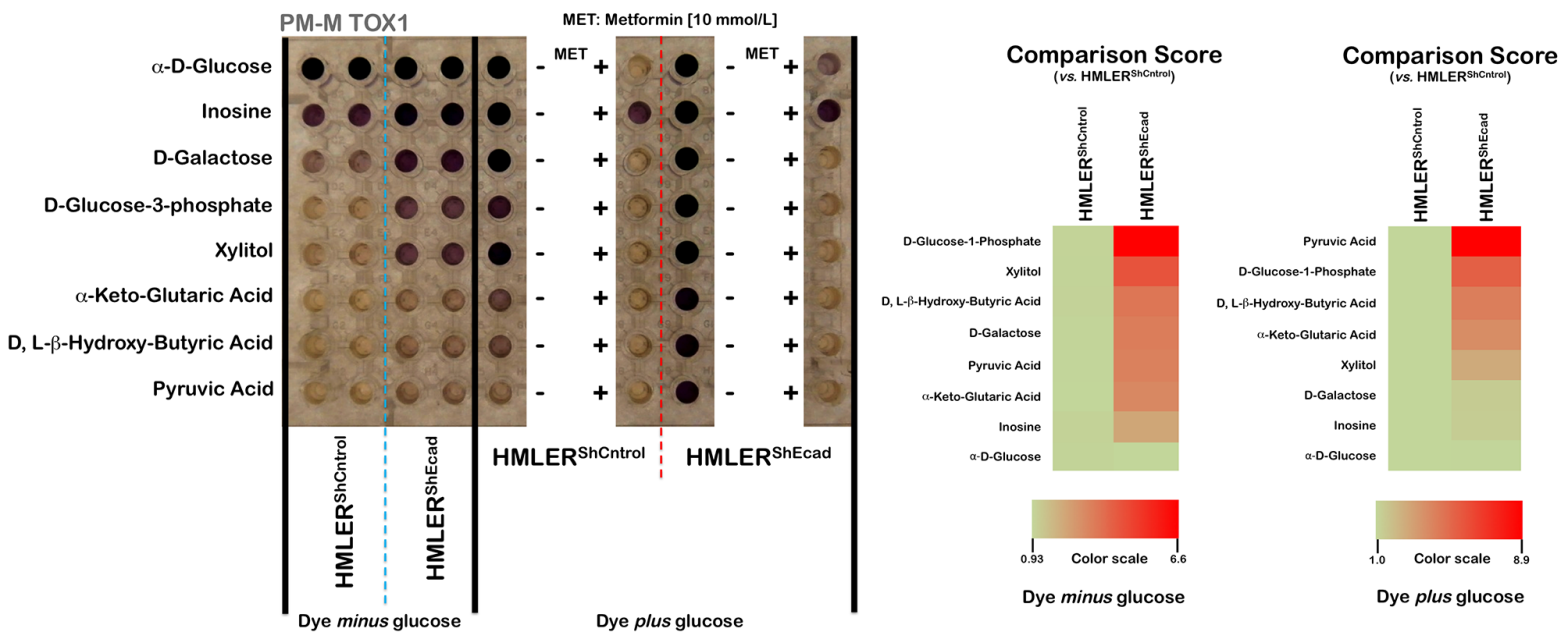
Alternative mitochondrial fuel of EMT-induced CS-like cellular states *Right panels.* Suspensions of non-CS HMLER^shCntrol^ and CS-like HMLER^shECad^ cells in RPMI-1640 that lacked phenol red and glucose were dispensed into Biolog PM-M TOX1 MicroPlate (Biolog, Hayward, CA) wells whose rows contain different carbon/energy sources as indicated. After two days of incubation, Biolog Redox Dye Mix MB without or containing glucose was added, plates were incubated at 37 °C under air to assess dye reduction 6 h (Redox Dye Mix MA) and then photographed. The respective utilization of substrates to generate energy-rich NADH was measured as ODs at 590 nm. *Left panels.* Phenetic maps of the utilization patterns of mitochondrial substrates in non-CS HMLER^shCntrol^ and CS-like HMLER^shECad^ cells. Phenotypes that are lost are colored green and phenotypes that are gained are colored red; the exact relative values are given by a corresponding color as indicated at the color scales.

To further corroborate the above-mentioned findings suggesting that EMT-induced CS-like cellular states could cell-autonomously enhance the vectorial transfer of energy-rich nutrients from the extracellular microenvironment to the energy-producing catabolic pathways in CS-like cells, we used the PM-M TOX1 MicroPlate to re-assess the differential utilization of eight carbon substrates by cellular mitochondria that are fed into the electron transport chain at different points (Fig. 3). The hexose D-galactose is metabolized to NADH *via* mitochondrial activity, whereas α-D-glucose can bypass these mitochondrial functions. Glucose-1-phosphate is metabolized differently from glucose and galactose, whereas ribose-containing inosine and xylitol are both metabolized (albeit differently) *via* the pentose phosphate pathway. α-ketoglutarate directly enters the tricarboxylic acid cycle (TCA) cycle, whereas the ketones β-hydroxybutyrate and pyruvic acid enter the TCA cycle upon metabolism and linkage to coenzyme A.

When these 8 substrates were tested to probe the differential utilization of bioenergetic pathways to generate NADH using tetrazolium dye with omission of glucose (the so-called “substrate metabolism assay”), we first confirmed that CS-like HMLER^shEcad^ cells metabolized the sugar phosphate D-glucose-1-phosphate and the monosaccharide D-galactose faster (approximate 7-fold and 3.5-fold increases, respectively) than HMLER^shCntrl^ parental cells. Whereas non-CS HMLER^shCntrl^ parental cells were fully unable to generate energy-rich NADH from the non-fermentable sugar xylitol, xylitol was found to be a very efficient energy source for the CS-like HMLER^shEcad^ cells (approximate 4.5-fold increase). CS-like HMLER^shEcad^ cells notably acquired the ability to efficiently generate energy-rich NADH (approximate 3.5-fold increase *versus* HMLER^shCntrl^ parental cells) from D, L-β-hydroxy-butyric acid, a ketone body that is produced during ketosis and used as an energy source by the brain under conditions of low blood glucose. α-keto-glutaric acid, one of the two ketone derivatives of glutaric acid, was also catabolized by CS-like HMLER^shEcad^ cells but not by HMLER^shCntrl^ parental cells.

When Biolog Redox Dye MB, which contains glucose, was added to detect the metabolic activity of the cells that remained viable after a 48-h incubation with the 8 different carbon substrates (the so-called “cell viability assay”), a highly divergent pattern was again observed between non-CS HMLER^shCntrl^ parental cells and CS-like HMLER^shEcad^ cells. Whereas CS-like HMLER^shEcad^ cells already catabolized pyruvate, the end product of glycolysis, approximately 3-fold more rapidly than HMLER^shCntrl^ parental cells in the absence of exogenous glucose, this capability was enhanced further in the presence of exogenous glucose (approximate 9-fold increase *versus* HMLER^shCntrl^ parental cells). Intriguingly, re-addition of exogenous glucose was not sufficient to rescue the very low levels of catabolism observed in non-CS HMLER^shCntrl^ parental cells after a 48-h exposure to the ketones pyruvic acid, D, L-β-hydroxy-butyric acid, and α-keto-glutaric acid. Conversely, exogenous glucose supplementation notably augmented the catabolic generation of energy-rich NADH in CS-like HMLER^shEcad^ cells, regardless of the nature of the carbon substrates employed.

To further study the mechanistic aspects of these dramatic changes observed in the dye-reduction assay, we tested non-CS HMLER^shCntrl^ parental cells and CS-like HMLER^shEcad^ cells in the PM-M TOX1 MicroPlate in the presence of high concentrations of the mitochondrial poison metformin. The re-addition of glucose was apparently insufficient to rescue the cells from the cytotoxic effects of metformin, which completely blocked dye reduction by substrates that would presumably be oxidized mitochondrially but partially rescued the production of NADH from inosine.

## The nutritional phenotype for the EMT-induced CS-like cellular states

A new perspective in cancer research. Universal phenotyping techniques that could discriminate among the various dynamically co-existing cellular states in heterogeneous cancer tissues may radically amend the current perception of cancer disease treatment [33-35]. Such techniques are a crucial research theme for CS cell-cellular state identification and monitoring because carcinoma aggressiveness derives not from the pre-existing CS cell content but rather from the proclivity of tumor tissues to generate new CS cells from non-CS cell populations [36-38]. A crucial challenge for the application of functional genomics to CS cells is the identification of genotypes associated with a specific phenotype. However, in contrast to well-established genotyping platforms, standard quantitative phenotyping methods are currently not available. Because normal and neoplastic epithelial cells have been shown to possess sufficient plasticity to re-enter the stem cell state *via* the interpretation of multiple microenvironmental signals [39-45], we recently reasoned that certain extracellular nutrients may be major determinants of the maintenance and/or expansion of the metabolic states characteristic of a CS-like cellular state. A comprehensive evaluation of the phenotypic variations among non-CS and CS-like isogenic cells based on a single-assay metabolic profile of several hundred nutrient sources may thus constitute a novel approach to the precise identification of CS-like cellular states and a starting point for uncovering the microecological nutritional niches of CS cells. In this scenario, we envisioned that PMM (Phenotype MicroArrays for Mammalian Cells) technology, which has been frequently used to analyze bacterial, yeast, and fungal mutant strains [28-32], would be well suited to a nutrient-response profile assessment of human CS-like cells. Indeed, the use of this standardized high-throughput metabolic phenotyping platform in the present study provided us with the first nutritional phenome for the EMT-driven CS-like cellular state with regard to energy-producing pathways.

Even at a gross level, PM-based global nutritional profiling immediately suggested important physiological consequences of acquiring a CS-like cellular state, as evidenced by the sole and specific loss of the epithelial marker E-cadherin. Both the qualitative and quantitative metabolic differences for the 367 substrates provided a simple but highly informative metabolic characterization that reflected the singular bioenergetics of acquired stemness in a cancer cell population. First, the CS-like HMLER^shEcad^ cells were much more metabolically active under both non-starved (28 additional energy-generating substrates) and starved (42 additional energy-generating substrates) conditions than their non-CS HMLER^shCntrol^ isogenic counterparts. Second, the types of bioenergetic fuels that could be employed by EMT-driven CS-like cells strictly corresponded to the availability of nutrients and energy sources in the extracellular milieu. For example, whereas an enhanced ability to generate energy from alanine and alanine-containing peptides appeared to be a metabolic property acquired by CS-like cells regardless of the microenvironmental condition (starved or non-starved), starved CS-like HMLER^shEcad^ cells exhibited a notable reduction in their ability to catabolize glutamate and glutamate-containing peptides, an acquired ability that was initially discovered in non-starved CS-like HMLER^shEcad^ cells. Alternatively, starved CS-like HMLER^shEcad^ cells rapidly acquired the ability to generate energy-rich NADH from glutamine and glutamine-containing peptides. Because tetrazolium dye reduction through nitrogen source utilization is measured indirectly via a linkage with carbon energy metabolism, these findings indirectly revealed that the coordination of NADH-producing metabolic pathways was drastically altered in EMT-driven CS-like cellular states relative to non-CS cellular states in an otherwise isogenic background.

## The nutritional phenome of CSCs: A new perspective on the “reverse Warburg effect”

A landmark study reported by Sonveaux *et al.* in 2008 [46, 47] suggested a “symbiotic relationship” among cancer cells in the tumor microenvironment where cancer cells distal to a blood vessel would be deprived of oxygen by cancer cells located proximal to the blood vessel. These distal hypoxic cells would then exhibit a different metabolic profile than the proximal cells and, indeed, hypoxic cells distal to the blood vessel have been shown to convert glucose to lactate, which could then be imported into aerobic cells and converted to pyruvate for mitochondrial oxidation. This model has been recently extended by the Lisanti group to suggest that the Warburg effect occurs in stromal cells rather than in cancer cells that feed off of stromal cell-generated lactate; this is in contrast to normal differentiated cells, which rely primarily on mitochondrial oxidative phosphorylation (OXPHOS) to generate the energy needed for cellular processes, as most cancer tissues instead rely on glycolysis even in the presence of sufficient oxygen. The provocative view submitted by the Lisanti group established that in cancer cells, mitochondrial OXPHOS can induce oxidative stress in adjacent fibroblasts (cancer-associated fibroblasts [CAFs]) *via* H_2_O_2_ and reactive oxygen species (ROS), thus resulting in the onset of an autophagic phenotype in CAFs. This autophagic phenotype subsequently leads to a loss of mitochondria *via* mitophagy and forces CAFs to undergo aerobic glycolysis (“reverse Warburg effect”) [48-65]. The increased aerobic glycolysis resulting from enhanced mitochondrial turnover in stromal cells generates excessive lactate, pyruvate, and other ketones, which are secreted into the intracellular space. The products of aerobic glycolysis are then re-used by cancer cells for OXPHOS, resulting in an increased mitochondrial mass. Importantly, the utilization by cancer cells of the high-energy autophagic stromal metabolites pyruvate, lactate, and ketones may increase the transcriptional expression of gene profiles normally associated with stemness, including genes commonly upregulated in embryonic stem cells. In addition to the resulting efficient net energy transfer from the tumor stroma to epithelial cancer cells in a unilateral and vectorial manner, this host-parasite intercellular cooperation between the tumor stroma and the epithelial cancer cells, which has been designated “the autophagic tumor stromal model of cancer cell metabolism”, “battery-operated tumor growth”, “stromal-epithelial metabolic coupling”, and the “reverse Warburg effect”, may therefore promote the dynamic appearance of a CS-like cellular state, thus resulting in a significant decrease in patient survival. Overall, the literature strongly suggests that cancer cells cannot effectively use ketones for fuel. Indeed, mostly in animal models of malignant glioma, ketogenic diets have been suggested as potential non-toxic treatments or adjuvant therapies to standard care for patients with systemic metastatic disease [66-70]. As there is apparently no known metabolic pathway by which fibroblasts can produce ketone bodies from glucose and as it is widely accepted that ketone bodies are produced nearly exclusively via the β-oxidation of fatty acids in hepatocytes, the “reverse Warburg effect” has been viewed as an artifactual phenomenon that occurs solely in the genetically engineered co-culture system used by the Lisanti group because it does not account for many observations that support cell-autonomous changes in cancer cell metabolism and excludes the possibility that cancer cells could utilize ketone bodies as efficient energy substrates.

**Figure 4:**
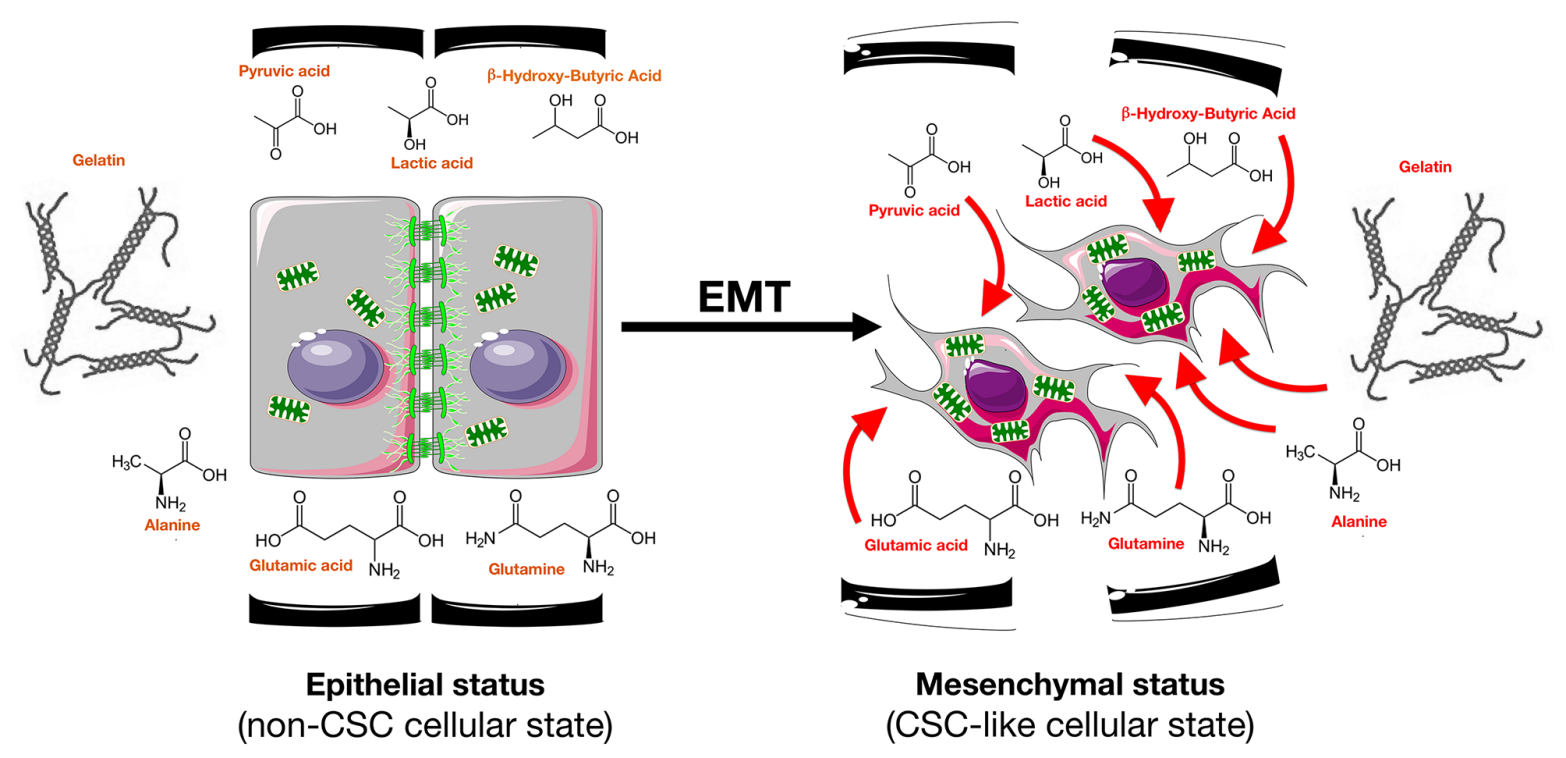
The nutritional phenotype of EMT-induced CS-like cellular states EMT-induced cancer stemness activates a metabolic infrastructure enabling the usage of extracellular, high-energy nutrients such as glycolysis end products (pyruvate, lactate) and *bona fide* ketone bodies (β-hydroxybutyrate) to support mitochondrial energy production. Metabolic reprogramming might constitute an efficient adaptive strategy through which pre-programmed EMT-CS-like phenotypes would rapidly obtain an advantage in hostile conditions such as nutrient starvation following the inhibition of tumor angiogenesis.

We now challenge the above-mentioned views by revealing that the acquisition of stemness traits by cancer cells undergoing EMT is sufficient to cell-autonomously enable the vectorial transfer of energy-rich nutrients from the extracellular microenvironment to energy-producing catabolic pathways in the CS-like cells. We present the first evidence for the existence of a cell-autonomous “reverse Warburg effect” where CS cell reprogramming pre-activates energy-producing pathways that can efficiently metabolize *bona fide* ketone bodies such as β-hydroxy-butyric acid *in vitro* (Fig. 4). Furthermore, the drastic increase in the ability of cells with EMT-induced CS-like cellular states to take up not only ketone bodies but other high-energy metabolites such as lactate [71] and, more importantly, pyruvate from the extracellular milieu and use these metabolites to feed mitochondrial energy production, especially upon starvation, is notable. In addition, cells with EMT-driven CS-like cellular states appear to acquire the ability to supra-additively generate energy-rich NADH upon re-exposure to an adequate source of exogenous glucose, regardless of the specific disposable nutrient source in the extracellular milieu. Cancer tissues are known to adapt to tumor microenvironment components such as hypoxia, nutrient deficiency, acidosis, and reactive oxygen species *via* altered cancer metabolism and CSC enrichment. Thus, long-term hypoxia and nutrient starvation contribute to tumor aggressiveness and recurrence because hypoxic and nutrient-starved tumor microenvironments suppress cell cycle progression but enrich non-proliferating CS cells [72-78]. In this scenario, the response to oxygen or nutrient deficiency and acidosis can be attained via metabolic alterations to glycolysis, glutamine metabolism, and other metabolic pathways. We now provide evidence that the EMT-driven switch from a non-CS- to a CS-like cellular state results in the pre-activation of a metabolic infrastructure that permits vectorial energy transfer from a broader range of extracellular nutrients, including high-energy metabolites such as pyruvate, lactate, and ketones, under stressful microenvironmental conditions. Metabolic reprogramming may thus constitute an efficient adaptive strategy through which these pre-programmed CS-like cellular states would rapidly obtain an advantage in hostile milieus such as those that follow the inhibition of tumor angiogenesis, a state wherein heterogeneous cancer cell populations are transiently exposed to both hypoxia and nutrient starvation; this state is known to stimulate tumor aggressiveness. The metabolic reprogramming of EMT-driven CS-like cellular states can elicit rapid adaptations to the metabolic tumor microenvironmental milieu that are likely responsible for the failures of antiangiogenic therapy with respect to metastatic tumors. Moreover, because alterations to the appropriate balance of fuels and/or signal transduction pathways that handle nutrient utilization have been suggested to underlie the cancer predisposition associated with metabolic diseases such as diabetes and obesity, our current findings offer a new perspective regarding how whole-body metabolism may directly interact with CS cells metabolism and therefore may better define these risks; for example, lactic acidosis and ketoacidosis could be viewed as systemic pre-neoplastic/pre-metastatic and possibly EMT-CS cell-enriching conditions [79-81].

## From nutritional phenotypes of EMT-CS-like cells to systemic “metabolic nichotherapies”

Our current results provide additional insights into the commonly oversimplified view of cancer metabolism. Global nutrient utilization analyses can offer a previously unrecognized complement to the phenotypic characterization of highly plastic CS-like cellular states, and it may provide a better understanding of the “genotype-phenotype” maps with respect to cancer pathogenesis and metastasis. A definitive elucidation of the metabolo-phenomic maps of CS cells may reveal an unforeseen route to the development of therapies against not only the intrinsic metabolic energy-generating machinery of CS cells but also their nutritional niches. The emerging discipline of nutritional genomics or nutrigenomics includes both the study of the effects of diet on an individual's gene activity and health and the study of how genetic composition affects nutrient metabolism [82-85]. Through an understanding of the unique roles of specific nutrients and their possible roles in boosting EMT-CS-like cell phenotypes, it might be possible to customize “smart foods” or systemic “metabolic nichotherapies” tailored to the specific nutritional phenomes possessed by cells with CS-like cellular states. Our findings also present a new CS cells-centered mechanistic perspective on the current investigations of dietary supplements and strict glucose control as adjuncts for managing the cachexia associated with excess tumor nutrient consumption. Moreover, simultaneous evaluations of the human plasma levels of energy-rich nutrients such as lactic acid, pyruvic acid, and 3-hydroxybutyric acid to facilitate the clinical monitoring of lactic acidosis and ketone body formation may provide a new strategy for predicting the whole-body metabolic regulation with respect to cancer risk and EMT-CS-like cellular phenotype-based metastatic progression. Nevertheless, because new techniques such as multi-isotope imaging mass spectrometry permit the high-resolution tracking of heavy isotope-labeled molecules upon utilization by specific types of cells [86, 87], the unique metabolic fluxes generated by the catabolic energy-producing machinery of CS cells could be implemented in a novel manner to monitor the spatiotemporal distributions and functionality of CS cellular states in real time.

## Metabolic phenomics of CS Cells

A practical corollary. As a practical corollary of this perspective it is necessary to bear in mind that we have taken advantage of the landmark Weinberg observation that breast cancer cell populations experimentally induced into an EMT drastically increase the proportion of CS-like cells [26, 27]. V12H-Ras-transformed derivatives of immortalized mammary epithelial cells (HMLER) driven to undergo the EMT by E-cadherin knockdown (HMLER^shECad^) exhibit characteristics of proliferative CS-like cells or cancer stemloids [26, 27, 88], which has been successfully exploited as a valuable screening method for preliminarily identifying agents with specific toxicity towards proliferating CS-like cells with stemness properties [27]. We confirmed that HMLER^shECad^ cells acquire a mesenchymal breast cancer phenotype and display an increase in the proportion of cells with a CD44^high^CD24^low^ marker profile [89], which was originally associated with human mammary CS cells (approx. a 10-fold increase) when compared with HMLER^shCntrl^ cells. HMLER^shECad^ cells also showed a dramatic increase in tumorsphere-forming ability relative to HMLER^shCntrl^ cells, which mostly failed to form *bona fide* mammospheres, an *in vitro* assay that gauges stemness (data not shown). Using a colorimetric assay extremely simple to perform, which does not require any prior knowledge of the energy pathways present and active in a given cell line, our current execution of the PM device with the Weinberg's conceptual modeling of EMT-induced CS-like cells clearly exemplifies the usefulness of the PMM technology for researchers wanting to study metabolic pathways activities preferentially owned by CS cells.

While metabolomic assay of pool levels might be better for detecting the presence of pathways and detecting the site of metabolic pathway blockages and the use of quantitative isotope tracking might be better to measuring pathway fluxes in CS-like cellular states, the PMM technology might provide researchers with a simple tool for a global perspective of the range with advantages for measuring *in vivo* metabolic fluxes and regulation of multiple energy-producing pathways, thus enabling a wide range of rapid studies relating tumor genotype to CS cells phenotypes. However, although EMT-induced CS-like cells have been evidenced in many cancer types, it is certainly a simplistic view to consider equivalent the *bona fide* CS cells, which is able to support the growth and heterogeneity of an entire tumor, with the ability of differentiated tumor cells to generate, through EMT, proliferating CS-like cell populations. Cancer stemness must be viewed as a multifaceted evolutionary hallmark of selected cancer cells endowed with the most competitive properties (*e.g.,* immortality, dormancy, chemo- or radioresistance, and EMT) resulting from several epigenetic mechanisms and signaling programs. In this complex scenario in which cancer stemness is an acquired and reversible trait that is quantitative rather than qualitative and that results from a stochastic rather than deterministic process, we acknowledge that the incorporation of robust metabolic phenomics, *i.e.* the systematic acquisition and objective documentation of cancer metabolic data at the level of CSC cellular states, might revolutionize the advancement of CSC-related cancer precision medicine. Because CS cellular states should involve changes in the capacity (enzyme abundance) and kinetics (enzyme activity) of certain metabolic nodes that might generate CS cell-associated onco-metabolites or metabolomic flux imprints, such a unambiguous, qualitative and quantitative phenomic representation of the CSC metabo-phenome will require a systematic combination of biology, biochemistry, genetics, metabolomics, fluxomics, and mathematical approaches. Nevertheless, the preliminary generation of carbon and nitrogen utilization “phenetic maps” upon the acquisition of CS-like cellular states, such as those presented here, might certainly accelerate our current speed of knowledge in the unique metabolic infrastructure of CS cells.
